# Selection of effective manufacturing conditions for directed energy deposition process using machine learning methods

**DOI:** 10.1038/s41598-021-03622-z

**Published:** 2021-12-17

**Authors:** Jong-Sup Lim, Won-Jung Oh, Choon-Man Lee, Dong-Hyeon Kim

**Affiliations:** 1 School of Smart Manufacturing Engineering, Changwon National University, Changwon, 51140 Republic of Korea; 2 Department of Mechanical Engineering, Changwon National University, Changwon, 51140 Republic of Korea; 3 Mechatronics Research Center, Changwon National University, Changwon, 51140 Republic of Korea

**Keywords:** Mechanical engineering, Metals and alloys

## Abstract

In the directed energy deposition (DED) process, significant empirical testing is required to select the optimal process parameters. In this study, single-track experiments were conducted using laser power and scan speed as parameters in the DED process for titanium alloys. The results of the experiment confirmed that the deposited surface color appeared differently depending on the process parameters. Cross-sectional view, hardness, microstructure, and component analyses were performed according to the color data, and a color suitable for additive manufacturing was selected. Random forest (RF) and support vector machine multi-classification models were constructed by collecting surface color data from a titanium alloy deposited on a single track; the accuracies of the multi-classification models were compared. Validation experiments were performed under conditions that each model predicted differently. According to the results of the validation experiments, the RF multi-classification model was the most accurate.

## Introduction

**Figure 1:**
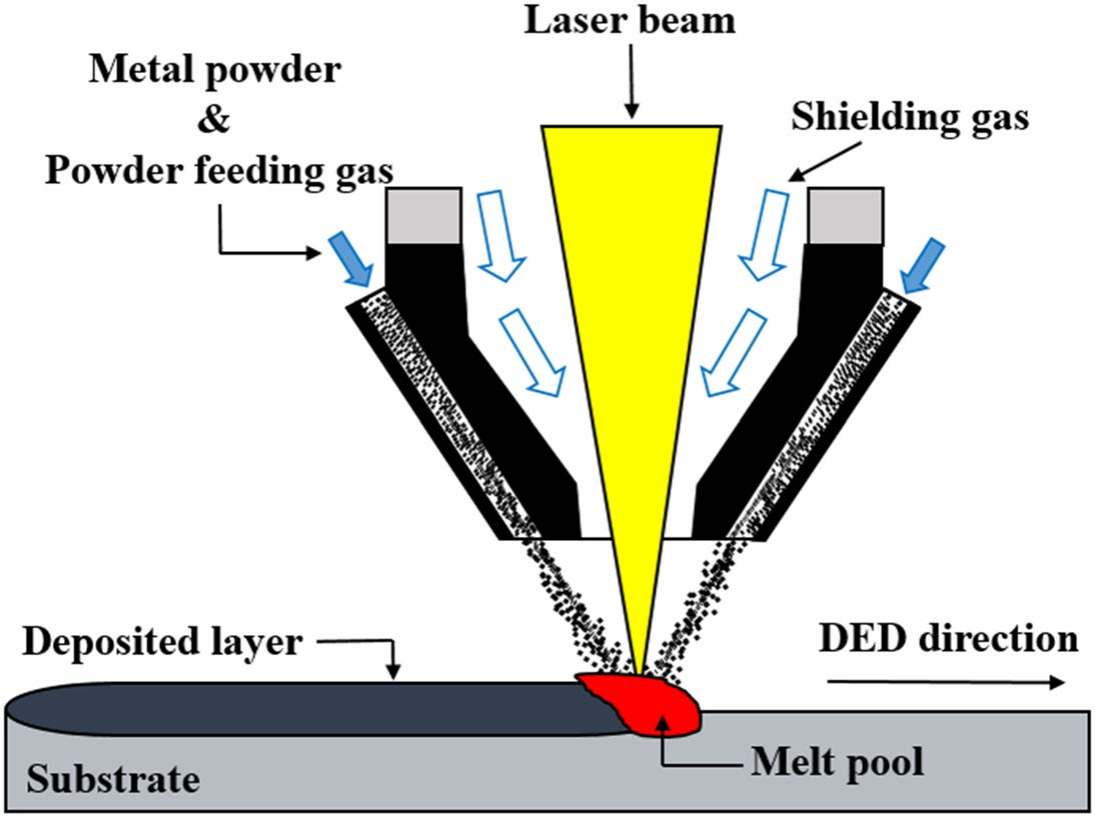
Diagram of laser powder direct energy deposition.

Metal additive manufacturing (AM) technology has emerged in the aircraft, automobile, and shipbuilding industries, and can realize designs that are not possible using conventional manufacturing methods^1^. Metal AM technologies can be classified into powder bed fusion (PBF) and direct energy deposition (DED) processes. The PBF process uses thermal energy (laser or electron beam) to selectively fuse regions of a powder bed, layer by layer, allowing the manufacture of complex shapes. The DED process uses a metal wire or powder combined with a thermal energy source to directly deposit material onto a substrate, resulting in excellent mechanical properties such as strength and elongation^2,3^. The PBF process fills the bed with powder, whereas the DED process feeds powder or wire only to where it is deposited. The DED process has been widely applied in the repair, remanufacture, and functional coating of metallic components^4–6^. In this study, the laser powder DED process was used. A diagram of the laser powder DED process is shown in Fig. 1. Laser power, scan speed, and powder feeding rate are process parameters applicable to the DED process. Depending on these parameters, the product quality such as the deposit height and width of the single-track, adhesion to the substrate, and porosity. In the DED process, product quality is affected by process parameters and researchers have studied their effects^7,8^.

Machine learning (ML), a branch of artificial intelligence (AI), is widely used to improve process quality, optimize manufacturing processes, and reduce costs in AM research^9–12^. Supervised learning is the most widely used method in AM research, classification and regression have been actively applied. The main difference between classification and regression is whether the outcome to predict is continuous or not. For example, regression should be used to predict dimensional values with continuity and classification should be used to predict defects through images. To make good use of machine learning, it is important for users to accurately recognize the problem they need to solve and to choose the algorithm to use. Khanzadeh et al. compared the performance of supervised learning methods for porosity prediction^13^. Sreeraj and Kannan et al. used an artificial neural network (ANN) to predict the track dimensions for wire feed gas metal arc welding using a range of input process parameters^14^. Li et al. studied a deep learning-based process monitoring method and a quality identification method for the metal AM process^15,16^. Gaikwad et al. studied heterogeneous sensing and machine learning for single-track quality in laser powder bed fusion^17^. Feenstra et al. demonstrated the complex interactions and relationships between the parameters using artificial neural networks in DED processes for Inconel 625, Hastelloy X, and stainless steel 316 L^18^. Random forest (RF) and SVM are ML algorithm methods for classification. Zhan et al. studied the prediction of the fatigue life of deposited SS 316 L using an ANN, RF, and SVM^19^. Zhang et al. studied the PBF process using SVM and a convolutional neural network (CNN) to identify and classify the deposition quality level, and compared the classification accuracy^20^. Gobert et al. described the development and implementation of an in situ defect detection strategy for PBF using an SVM^21^. Aoyagi et al. proposed a simple method for constructing a process map for additive manufacturing using an SVM^22^. Many researchers have used machine learning for complex processes.

Titanium alloy is widely used in the aerospace and medical industries due to its excellent mechanical properties and corrosion resistance. Titanium alloy microstructure fabricated by DED is observed to have a range of as-deposited microstructures primarily classified as being basket-weave widmanstatten, or acicular or martensitic and consist of large prior-$\upbeta$ grains that grow epitaxially across subsequent build layers in DED^23^. The laser power and scan speed, the process parameters of this study, affect the amount of the cooling rate. The effects of cooling rate during additive manufacturing can change the resulting microstructure which affects mechanical properties.

The aim of this study is to obtain effective manufacturing conditions for a single-track DED process for titanium alloy powder. The laser power and scan speed were set as the process parameters. The deposited microstructure of titanium alloy made by AM is anisotropic due to the rapid solidification where the material is added in a layer-by-layer fashion. The deposited samples were labeled by surface color and used as training data for machine learning. Labeled samples were analyzed using cross-sectional view, hardness, microstructure, and component analyses, and the best deposition surface color was selected. Three prediction models were proposed using RF and SVM in machine learning methods. The results of the validation experiments confirmed the RF model as the best model. The proposed model can be used as an index to select effective manufacturing conditions for the DED process for titanium alloy powder.

## Methods and materials

### Classifiers

#### Random forest (RF)

*Decision tree* A decision tree is a predictive model used to effectively classify a dataset. The predictive model divides the dataset into smaller subsets to determine the best decision in the analysis process. However, as only one variable is considered at a time, there is a limitation in assessing the interaction between variables. An RF model appears to solve this problem.

*Bagging (bootstrap aggregating)* Bagging refers to an algorithm that creates multiple classifiers; the final classifier is decided by voting. Bootstrap sampling is a method that allows overlapping of some data in the dataset.

*Random forest classifier* An RF is a machine learning technique used to classify data. An RF is an ensemble method, a machine learning technology expressed as a forest composed of numerous decision trees. An RF is divided using the bootstrapping method. Decision tree classifiers sample data based on bagging, perform training, and make prediction decisions through voting. In this study, using the random forest classifier using the deposited surface color as training data, The surface color of the unexperimented area was predicted.

**Figure 2:**
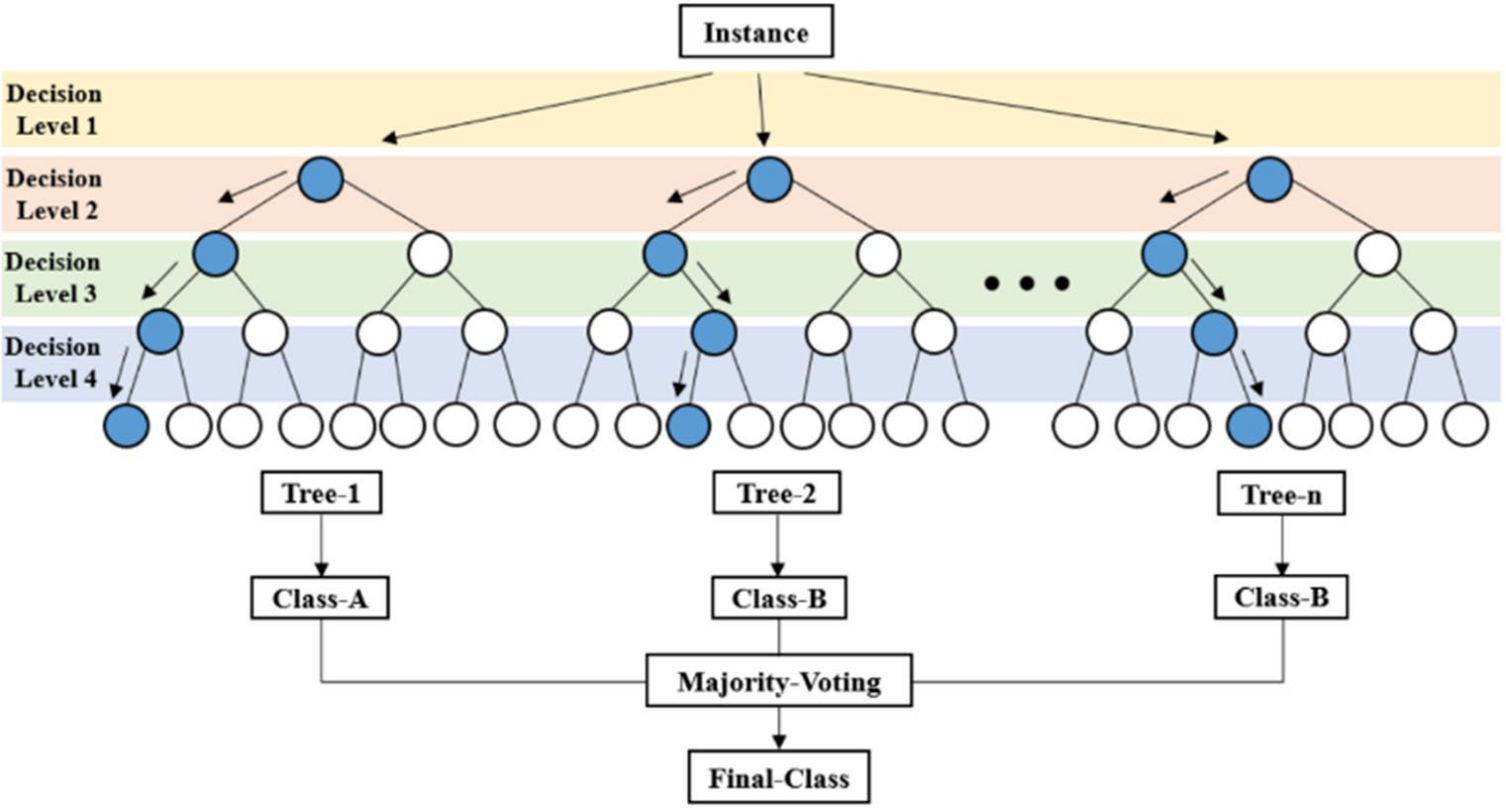
Functional diagram of random forest classifier^24^.

In an RF, the overfitting of the decision tree algorithm is reduced by combining multiple decision trees to obtain an accurate final decision^24^. A functional diagram of an RF classifier is shown in Fig. 2.

#### Support vector machine (SVM)

An SVM is a machine learning technique used to classify data. The concept of applying an SVM to pattern classification can be described as follows. First, the input vector is mapped linearly or nonlinearly into one feature space (possible at higher dimensions). The optimized linear segmentation is determined within the functional space from the first step. In some cases, data points that are not linearly separable are transformed using kernel functions to become linearly separable. The kernel methods map the input space data, which is a low dimension space, into a much higher dimensional feature space using a nonlinear mapping $\emptyset$. There is a highly effective trick for calculating inner products in the feature space using a kernel function.

1 $$ {\text{K}}\left( {x_{i} , x_{j} } \right) = \emptyset \left( {x_{i} } \right)^{T} \emptyset \left( {x_{j} } \right) $$

By using kernel functions, various types of nonlinear models in the original space could be constructed. The kernel functions used in this study are listed as follows.

1. Polynomial kernel function
  2a $$ {\text{K}}\left( {x_{i} , x_{j} } \right) = \left( {\left\langle {x_{i} ,x_{j} } \right\rangle } \right)^{d} ,\quad c > 0. $$
2. Radial-basis kernel function (RBF)
  2b $$ {\text{K}}\left( {x_{i} , x_{j} } \right) = \exp \left( { - \gamma \left\| {x_{i} - x_{j} } \right\|^{2} } \right),\quad \gamma > 0. $$
  where $\gamma$, c, and d are kernel parameters.

**Figure 3:**
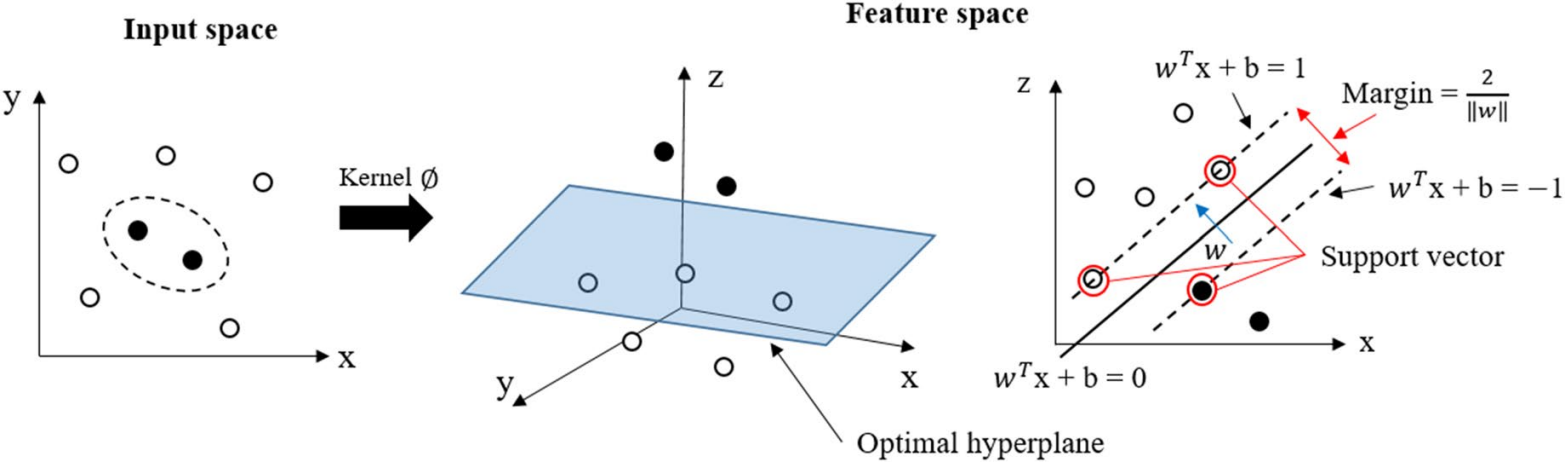
Functional diagram of support vector machine.

The input data are classified by selecting the appropriate hyperplane. The nearest point from the hyperplane is the support vector. As a classifier, the SVM finds a hyperplane in a high-dimensional space, which creates a maximum margin between the classes representing the longest distance between the closest data points. The input data are classified by selecting the appropriate hyperplane. The width of the margin is $\frac{2}{\left\| w \right\|}$, and when ${\text{w}}$ is minimum, the margin is maximum. When $\left\| w \right\|^{2}$ becomes minimum, ${\text{w}}$ becomes minimum, which is called SVM optimization. A functional diagram of the SVM is shown in Fig. 3.

### Procedure

**Figure 4:**
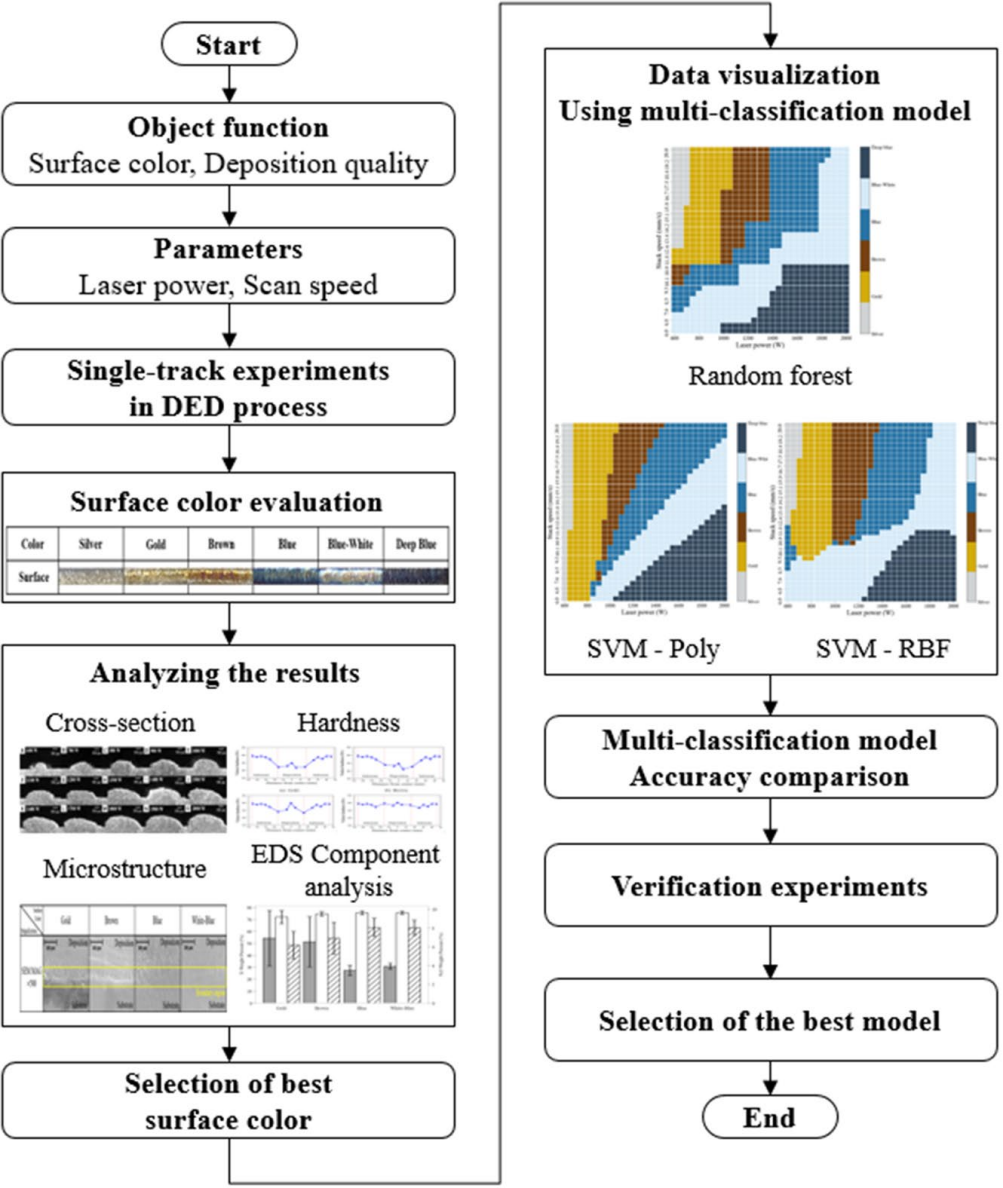
Flowchart of experimental procedure.

This section describes the experimental procedure flowchart. The objective function of the experiment was the deposition surface color and quality in the single-track titanium alloy DED process. The process parameters in the experiment were the laser power and scan speed. The experiment confirmed six surface colors including silver, gold, brown, blue, blue-white, and deep blue. The color was defined with the naked eye. Cross-sectional view, hardness, and EDS component analysis were conducted to determine the structural and mechanical properties of the deposition surface, and the best surface color was selected. Three multi-classification models were proposed using RF and SVM, and their accuracies were compared. The best model was selected through validation experiments. A flowchart of the experimental procedure is shown in Fig. 4.

### Experimental set-up

**Figure 5:**
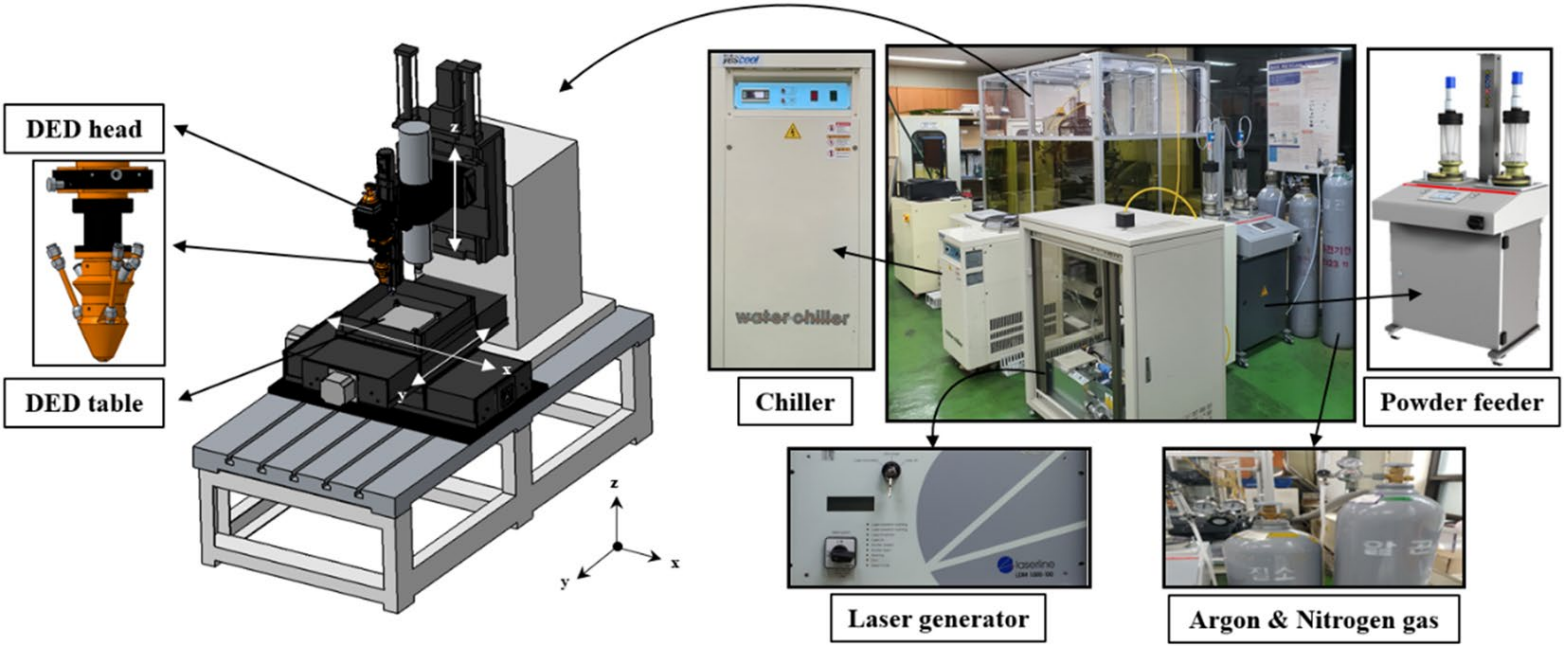
Experimental DED set-up.

In this experiment, a machine tool equipped with a high-power diode laser deposition head was used (Laytools, AK390). The maximum laser power was 2 kW at a wavelength of 980 nm. The laser focus was calibrated with a lens to a focal length of 198 mm and a laser beam diameter of 3 mm. Argon was used as the shield gas, and nitrogen was used as the powder-feeding gas. The powder-feeding gas supplied the metal powder to the melt pool using the 3-way powder carrying pipe of the additive head. A powder feeder (Oerlikon Metco, Twin 150) was used with a twin-feeder head system. The system quickly and stably controlled the powder feed rate. A cooling system with 780 W of power (Yescool, YRC-1A) was used to cool the equipment. The 3-axis stage (X, Y, Z) had a travel distance of 500 mm × 500 mm × 300 mm, a UMAC controller was used to control the 3-axis stage, the feeder, and the laser head. The DED experimental setup is shown in Fig. 5.

Titanium alloy powder by the commercial product of KOS Ltd. was used. The metal powder is produced by the gas atomization method. The chemical composition of the powder is presented in Table 1. The particle size was 45–150 μm^25^.

**Table 1:** Chemical composition of titanium alloy powder.

Element	Ti	Al	C	Fe	V	N	O	H
wt%	Bal.	6.5	0.026	0.15	4.3	0.003	0.18	0.001

## Results and discussion

### Single-track experiments and evaluation

The selection of process parameters is important in the DED process. The process parameters are the input values, and must be set prior to machine operation. The important parameters in the DED process are the laser power, scan speed, and powder feed rate. The accuracy and quality can be changed according to the values of each parameter. The process parameters are influenced by the material properties and the machine. Thus, studies have been conducted on the correlation between process parameters and output quality. Sampson et al. studied the effects of the powder mass flow rate and path velocity changes on the molten pool^26^ The increase in the height and width of the single track with an increase in the powder feed rate is affected by the laser power; it cannot be simply assumed that the melt pool width increases as the powder feed rate increases. In this experiment, the powder feed rate was set to 14 g/min, considering the laser power and conservation of powder. The scan speed was set to 6–20 mm/s, considering the working time of the DED process. The laser power was set to 600–2000 W, considering the laser specifications and the melting of the powder. The process parameters and ranges used in the experiments are presented in Table 2. Single-track experiments were conducted. The surface color appeared differently according to changes in the laser power and scan speed.

**Table 2:** Process parameter ranges used in experiments.

Process parameter	Test range
Laser power (W)	600–2000
Scan speed (mm/s)	6–20
Powder feed rate (g/min)	14
Argon gas flow (L/min)	25
Nitrogen gas flow (L/min)	5

**Figure 6:**
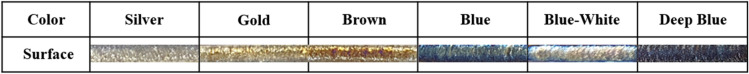
Deposition surface color results.

Titanium alloys are easily oxidized and nitrified. Reaction with oxygen is the most problematic, whereas nitrogen is generally considered negligible Due to the rate at which titanium alloy reacts with oxygen, there is a very small but constant oxide layer on the surface^27^. The formation of the oxide layer causes a color change on the surface of the titanium alloy. The color change in titanium alloy is a product of the oxidation layer^28^. In the titanium alloy DED process, a multi-colored deposition surface may be the result of oxidation and nitrification. A total of 135 process parameter sets were tested. The observed surface colors are shown in Fig. 6. The color threshold can be checked in Appendix A.

**Figure 7:**
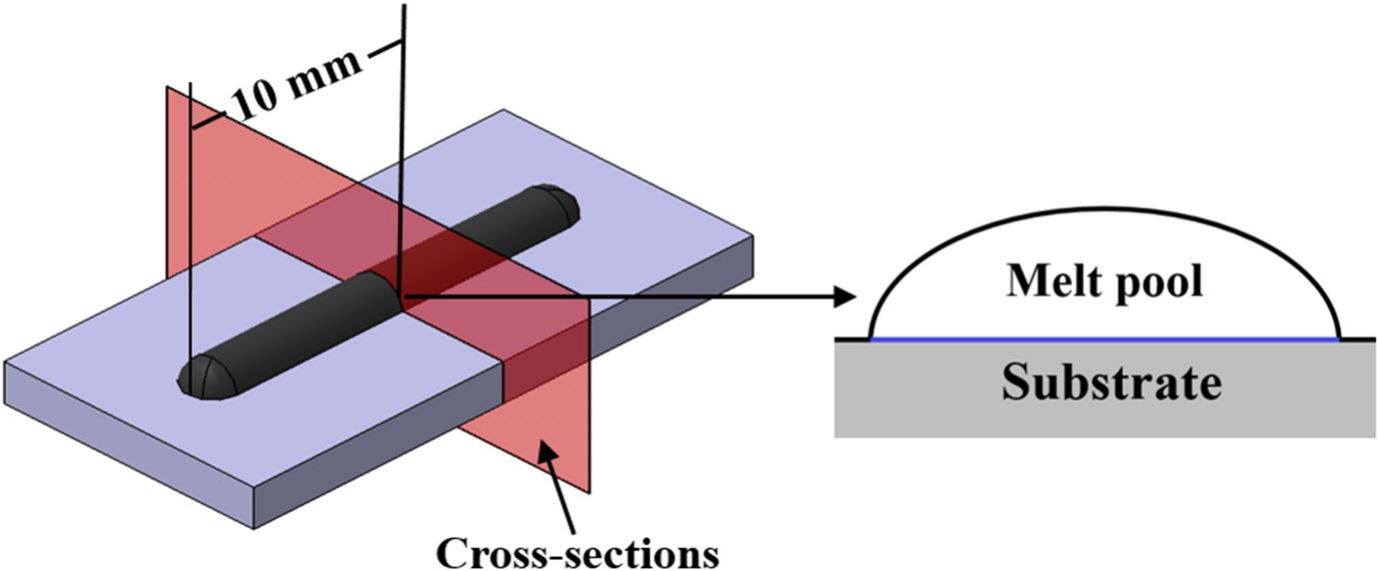
Diagram of single-track cross-section.

A single track of 20 mm was deposited onto a 15 mm thick titanium alloy substrate. The single-tracks were sectioned through wire cutting such that cross-sections perpendicular to the scan tracks were exposed. The cross-sections were located 10 mm from the beginning of the scan track. The cross-sections were imaged using an Olympus LEXT laser confocal microscope. A diagram of a single-track cross-section is shown in Fig. 7.

**Figure 8:**
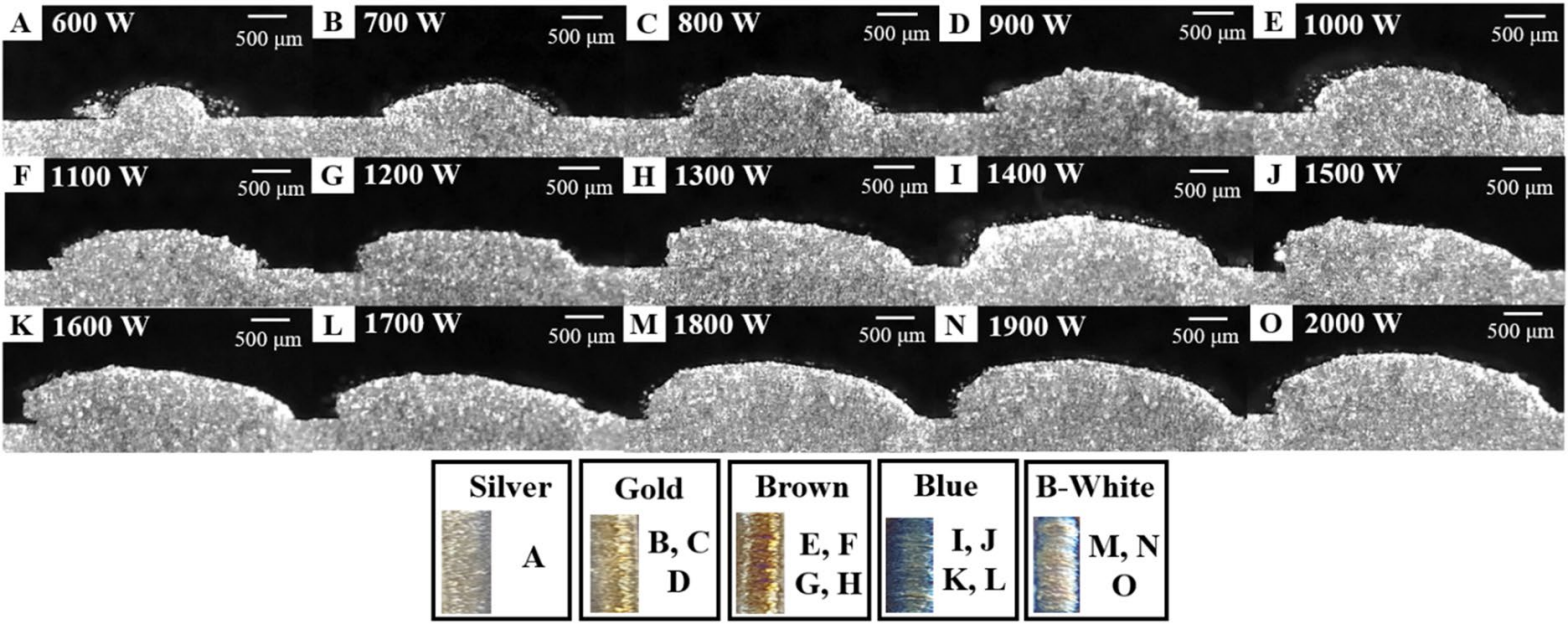
Titanium alloy deposited on substrate at constant scan speed of 14 mm/s with increasing laser power from 600 to 2000 W.

Cross-sectional view, hardness, and component analyses were performed to determine the structural and mechanical properties of the deposition surface. The cross-section was observed to assess the deposition quality according to the color. Figure 8 shows a cross-section while increasing the laser power after fixing the scan speed at 14 mm/s. From the analysis, the laser power increased the melt pool height, and the width increased. The colors of the deposition surface appeared in the order of silver, gold, brown, blue, and blue-white.

**Figure 9:**
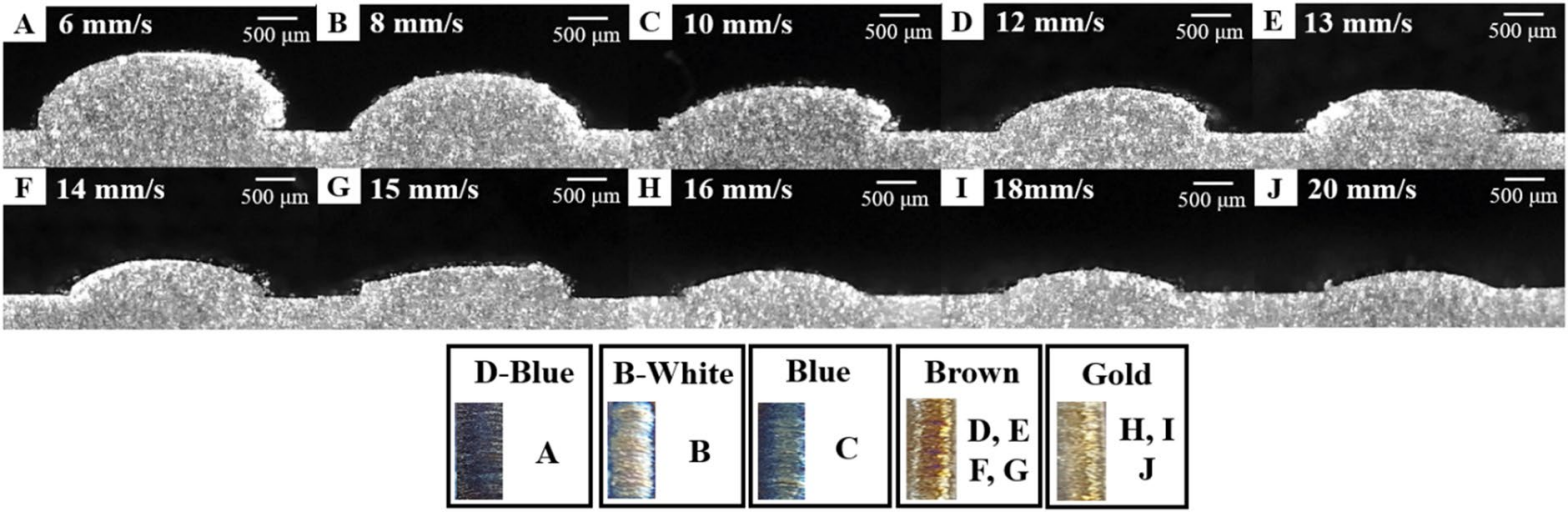
Titanium alloy deposited on substrate at constant laser power of 1000 W with increasing scan speed from 6 to 20 mm/s.

Figure 9 shows a cross-section while increasing the scan speed after fixing the laser power at 1000 W. From the analysis, the scan speed had a decreasing effect on the melt pool dimensions. The colors of the deposition surface appeared in the order of deep blue, white-blue, blue, brown, and gold.

**Figure 10:**
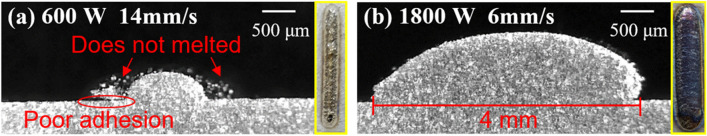
Instability of melt pool: (**a**) insufficient heat input; (**b**) excessive heat input.

Melt pool instability was confirmed in the deposition cross-sections. A scan speed too fast for the laser power resulted in insufficient heat input. With insufficient heat input, the surface color was silver. A high laser power and a slow scan speed result in excessive heat input. With excessive heat input, the surface color was deep blue. The instability of the melt pool is shown in Fig. 10. As explained in the experimental set-up, the laser spot is 3 mm. When the surface color was silver, a cross-section that did not reach 3 mm was observed. Also, as shown in Fig. 10a, parts that were not completely melted or did not adhere to the substrate were observed. When the surface color was deep blue (Fig. 10b), cross-sections of more than 3 mm were observed. In this case, it is excluded from the analysis because it is a disadvantage condition when multi-layer deposits and other shapes are made.

4 specimens with differnt surface colors were used for measurements. The specimens were hot-mounted using monting powder on the mounting press machine (NA-MA2031, Nanotech, Korea). And then, the specimens were polished on polisher with auto-head type (NA-P2000A, Nanotech, Korea). The final specimens for measurement were prepared by chemical etching after polishing.

**Figure 11:**
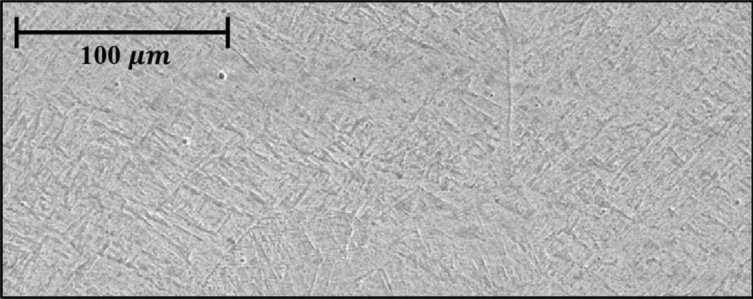
An example of the prior-β grain morphology in DED.

**Figure 12:**
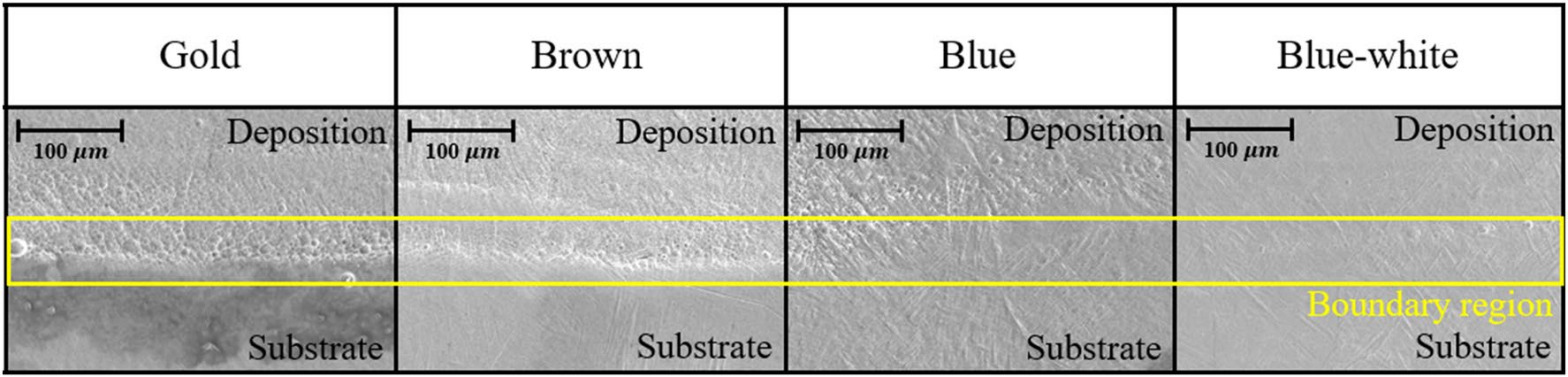
Boundary region microstructure according to deposition surface.

An example of the prior-b grain morphology in DED is shown in Fig. 11. The microstructure characteristics of the deposition according to the surface color were confirmed using an electron scanning microscope. There are four deposition surface colors in the analysis: gold, brown, blue, and blue-white. The boundary region microstructure according to the color of the deposition surface is shown in Fig. 12. The growth of the microstructure is affected by the cooling rate. The microstructure could not be grown due to the fast cooling rate by the process parameters of the fast scan speed and low laser power. As a result of observing the boundary region when the surface color was gold or brown, it was confirmed that the microstructure did not grow into large prior-β grains due to the fast cooling rate (low laser power, fast scan speed). When the surface color was blue, large prior-β grains and non-grown microstructures were observed together as the microstructure grew with a slower cooling rate than before. When the surface color was blue-white, most of the microstructures grew and were observed as large prior-β grains. We wanted to obtain a microstructure most similar to that of the substrate. Therefore the optimal microstructure condition was selected by blue-white.

**Figure 13:**
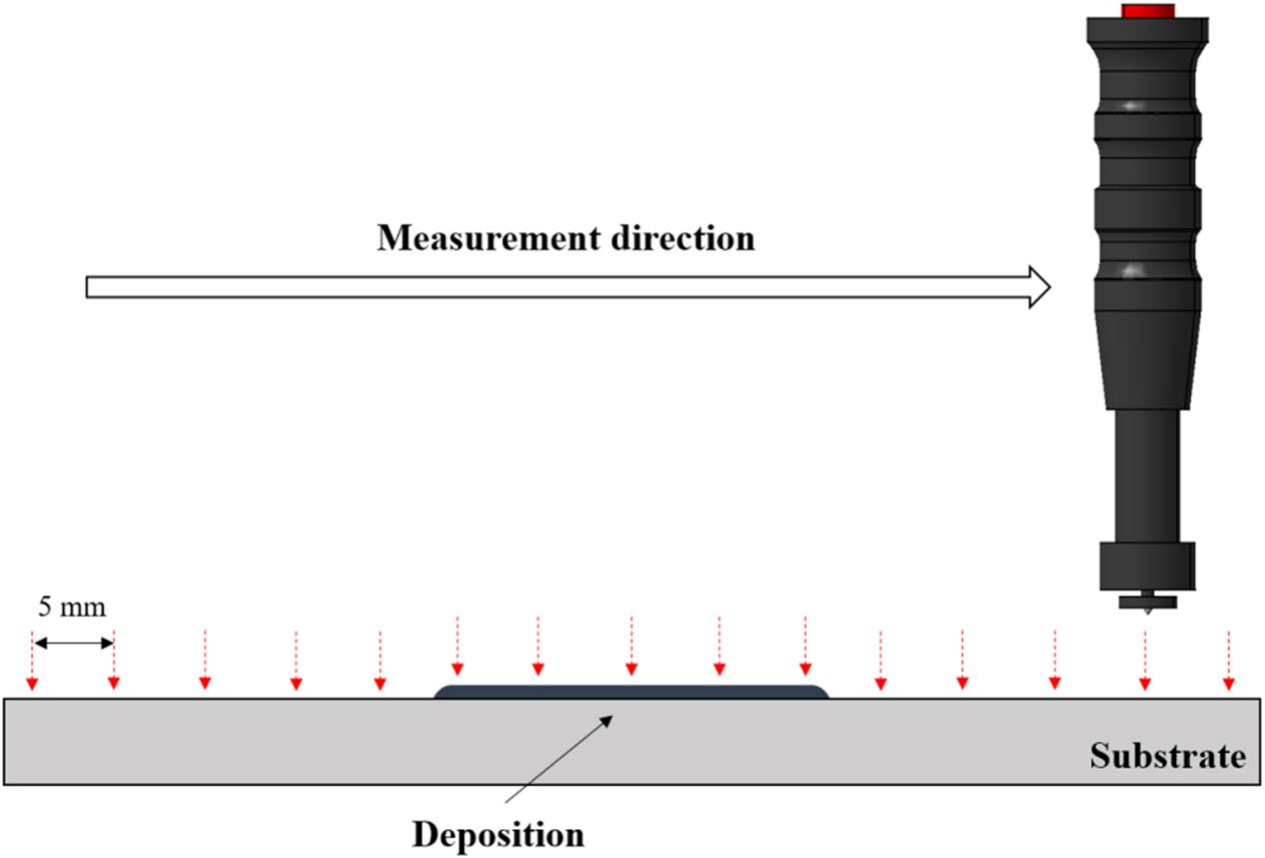
Hardness measurement diagram.

**Figure 14:**
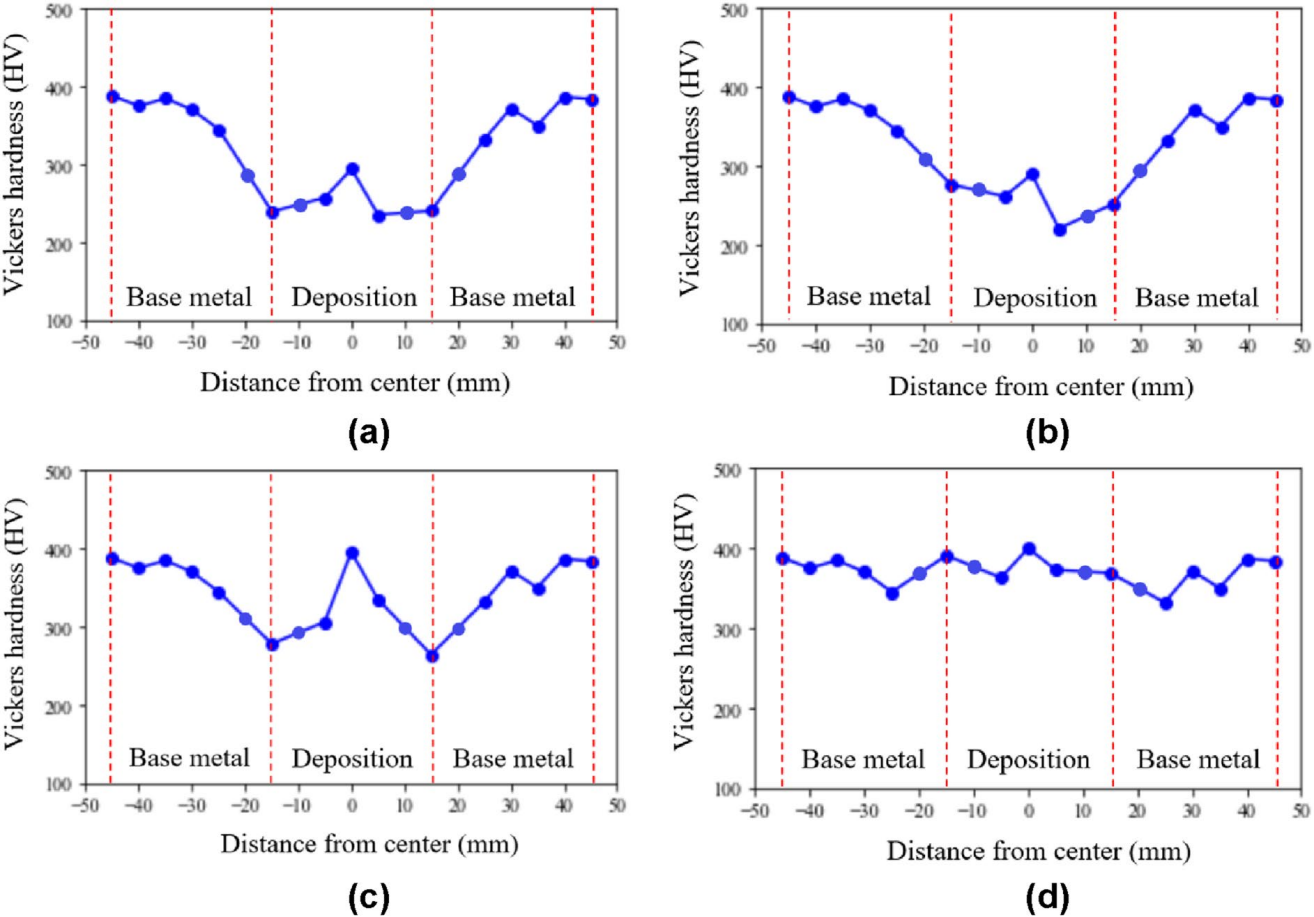
Hardness measurement results by color of deposition surface: (**a**) gold; (**b**) brown; (**c**) blue; (**d**) blue-white.

Hardness was measured using a rebound-type portable hardness tester manufactured from Mitutoyo. The hardness were measured at intervals of 5 mm in the deposit direction from a point 20 mm away from the deposition starting point. The hardness measurement diagram was shown in Fig. 13. Ten samples Hardness measurement results by the color of the deposition surface are shown in Appendix B. It was confirmed that the tendency of hardness was the same for each surface color. The hardness measurement results by deposition surface color are shown in Fig. 14. The Vickers hardness of the substrate was 350–400 HV. When the deposition surface color was gold or brown, the Vickers hardness was 200–300 HV. When the deposition surface color was blue, the Vickers hardness was 260–400 HV. When the deposition surface color was blue-white, the Vickers hardness was 360–410 HV. The hardness values tended to increase in the order of gold, brown, blue, and blue-white. A deposition surface color of gold, brown, or blue can be considered as a deposition defect, as the surface did not reach the hardness of the substrate. The best microstructure surface color indicates the best hardness.

**Figure 15:**
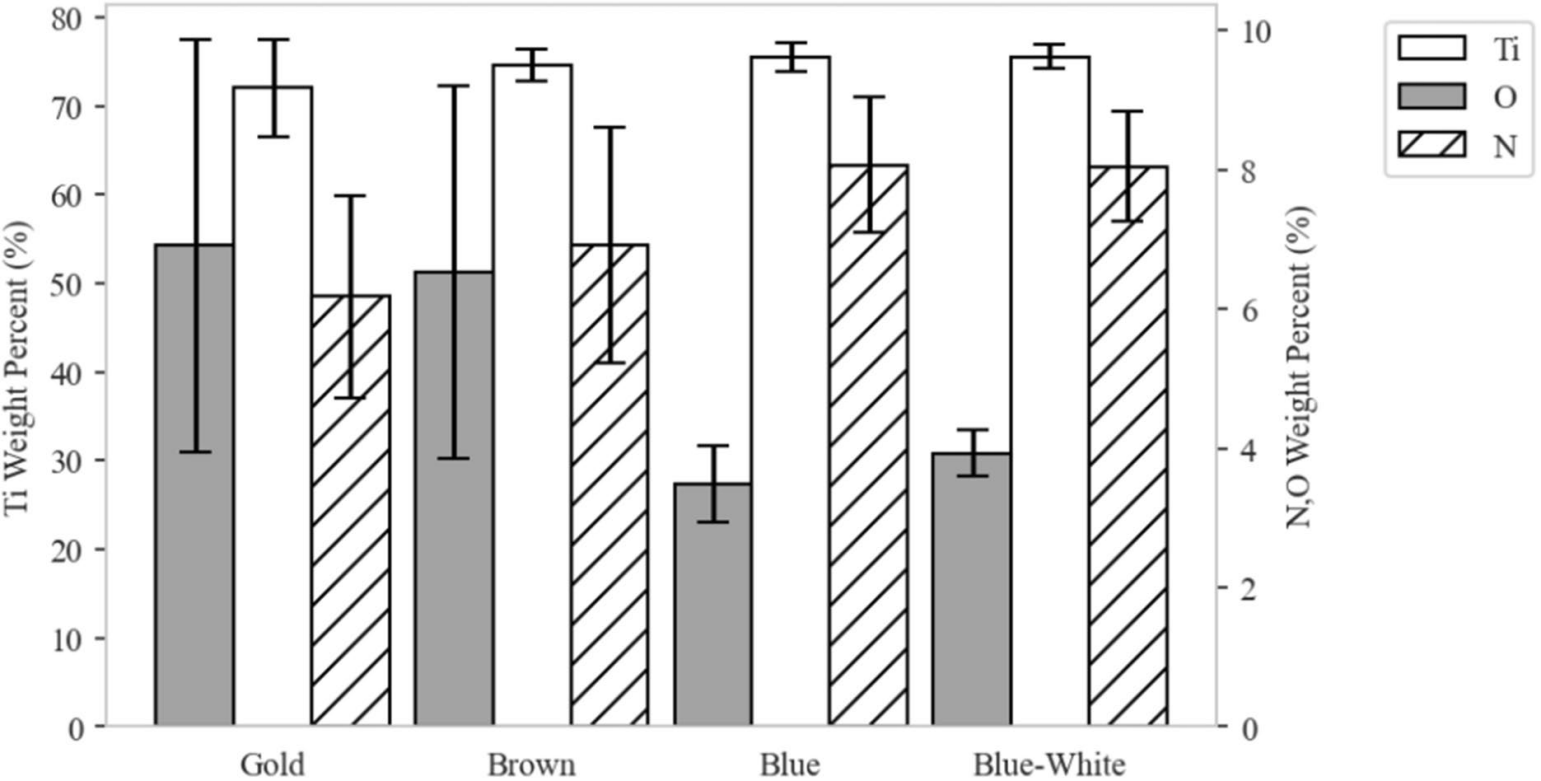
EDS component analysis results by color of deposition surface.

When the titanium alloy was deposited, it was confirmed that the microstructures were different depending on the surface color; a detailed EDS component analysis was performed. EDS component analysis was measured using a field emission scanning electron microscope (FE-SEM) MIRA II LMH model from TESCAN. The specifications of the microscope are shown in Table 3. Ten samples of EDS component analysis results by the surface color are shown in Appendix C. The results of the EDS component analysis by deposition surface color are shown in Fig. 15. For spot analysis, ten points were measured, and the average value was calculated. The elements used in the analysis were titanium, nitrogen, and oxygen; the average and maximum/minimum values of each element according to the deposition surface color are shown in the graph. When the deposition surface color was gold or brown, the oxygen content was greater. When the deposition surface color was blue or blue-white, the titanium and nitrogen contents were greater, and the oxygen content was smaller. From analysis of the structural and mechanical properties of the deposited surface colors, the blue-white surface color was the best.

**Table 3:** The specifications of the microscope.

Parameters	Specification
Magnification	× 4–1,000,000
H.V.	0.2–30 kV (0.1 kV step)
Resolution	SEI: 1.0 nm (30 kV), BEI: 2.0 nm (30 kV)
Internal size	230 mm Dia
Electron gun	High brightness Schottky emitter with EDS & EBSD

### Multi-classification model

Latin Hypercube Sampling (LHS) is one of the random sampling methods for selecting processes evenly over the entire sampling space, which is a method of randomly selecting and evenly distributing values from a defined distribution of each assumption. LHS is sampled more uniformly and consistently over the entire range. The experimental conditions were set using LHS. A total of 135 single-track experiments were performed; Python was used as a programming language. The scikit-learn library was used to perform ML calculations such as random forest and support vector machine. 108 were used as training data. The remaining 27 cases were used as test data to verify the classification model.

When using ML algorithms, it is necessary to adjust some variables, called hyperparameters, to find an accurate model. Hyperparameters must be set before the models are trained, and are critical for building accurate models. Both Random Forest and SVM are algorithms used for classification. SVM uses a kernel trick and has a fast computation seed and has various computation options depending on the kernel. RF can make very accurate predictions based on multiple decision trees. SVM doesn’t work well when you have a lot of samples. Also, you need to pay a lot of attention to setting hyperparameters. RF produces good results even with default settings without hyperparameter tuning. The hyperparameter values affect the performance of the model. The n_estimator is an important hyperparameter in an RF that determines the number of decision trees. When the number of decision trees increases, the performance of the model increases. An RF has many hyperparameters and requires significant time for tuning. The hyperparameter values affect the performance of the model. The n_estimator is an important hyperparameter in an RF that determines the number of decision trees. When the number of decision trees increases, the performance of the model increases. However, a greater n_estimator increases calculation time, eventually without improving performance. The n_estimator was set to 1000.

**Figure 16:**
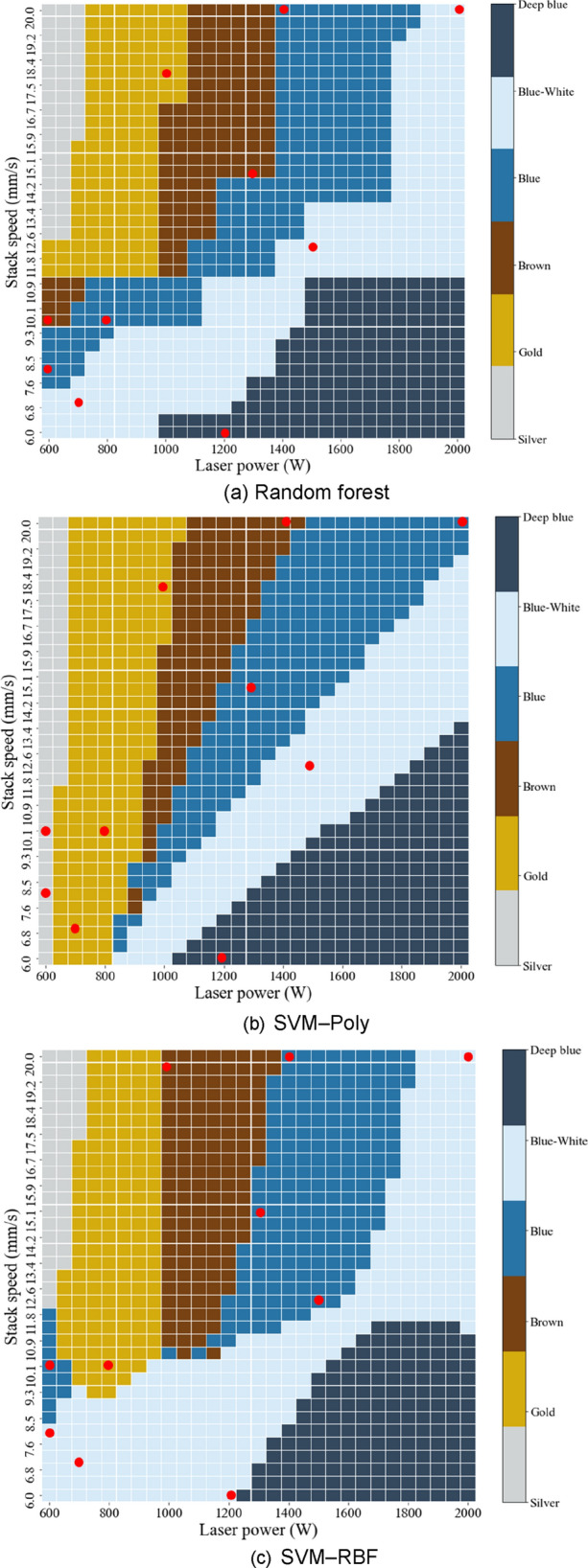
Proposed multi-classification models.

The performance of SVM could be improved by adjusting hyperparemeters under differnt noise levels, types of noise, target functions, and sample size^29^. The training data were mapped using kernel tricks such as a polynomial function (Poly) and a radial basis function (RBF) to classify the nonlinear data. The C parameter is an important hyperparameter in the SVM. When the model was under fitted, C was used to improve learning performance; when the model was over fitted, C was used to improve generalization performance. C was set to 10,000. The three multi-classification models are presented in Table 4. The RF model had the highest accuracy. A total of 1015 multi-classification cases were predicted; the three multi-classification models are shown in Fig. 16.

**Table 4:** Three multi-classification models.

Algorithm	Random forest	SVM–RBF	SVM–Poly
Hyperparameter	n_estimator = 1000	C = 10,000	C = 10,000
Accuracy	0.9643	0.8571	0.8214

The results of the validation experiments for the three models are shown in Table 5. Validation experiments were conducted for ten conditions that each model predicted differently. In Table 5, predictive and validation experimental accords are shown in bold. The conditions of the verification experiment are indicated by red points in Fig. 15. In ten validation experiments, the RF model yielded the most accurate predictions. The SVM model using the poly kernel yielded the lowest prediction accuracy.

**Table 5:** Validation experiment results for three models.

LP (W)	SS (mm/s)	RF	SVM–Poly	SVM–RBF	Validation experiment
600	8	**Blue**	Silver	Blue-white	Blue
600	10	**Brown**	Silver	Blue	Brown
800	10	**Blue**	Gold	Gold	Blue
1000	18	**Gold**	**Gold**	Brown	Gold
2000	20	**Blue-white**	Blue	**Blue-white**	Blue-white
1300	15	**Brown**	Blue	Blue	Brown
1200	6	**Deep blue**	**Deep blue**	Blue-white	Deep blue
700	7	**Blue-white**	Gold	**Blue-white**	Blue-white
1500	12	**Blue-white**	**Blue-white**	Blue	Blue-white
1400	20	**Blue**	Brown	**Blue**	Blue
Prediction success		10	3	3	

Significant values are in [bold].

## Conclusions

The purpose of this study was to select manufacturing conditions for a titanium alloy powder DED process using machine learning methods. Through analysis, it was confirmed that the blue-white surface was an effective manufacturing condition. Then, in order to select efficient manufacturing conditions RF and SVM multi-classification models were proposed. Three models were compared, and validation experiments were performed. The RF model was the best model that indicated the efficient selection of the blue-white manufacturing condition. The following conclusions were drawn from this study.

1. A total of 135 single-track experiments were performed with laser power and scan speed as the process parameters. Six surface colors were observed: silver, gold, brown, blue, blue-white, and deep blue. When the surface color was silver or deep blue, melt pool instability was observed.
2. The best deposition surface color was selected by analyzing the structural and mechanical properties; the blue-white surface color was the best.
3. Three multi-classification models using RF and SVM were proposed. Validation experiments were performed to compare the accuracies of the models; the RF model was the most accurate model. The RF model was the best model that indicated the efficient selection of the blue-white manufacturing condition.

Since there is a difference in the stacking quality depending on the process parameters, many experiments are required to find the optimal process parameters. When performing this, we propose a classifier using machine learning so that workers can select the blue-white condition, which is the best color presented in this paper. workers can select process parameters just by looking at the surface color. It allows researchers to efficiently select manufacturing conditions. The proposed model will be used for a multi-layer DED process in future research.

## Supplementary Information

Supplementary Tables.

## References

[1] Ren WJ, Mazumder J (2020). In-situ porosity recognition for laser additive manufacturing of 7075-Al alloy using plasma emission spectroscopy. Sci. Rep..

[2] Woo YY, Han SW, Oh IY, Moon YH, Ha W (2019). Control of directed energy deposition process to obtain equal-height rectangular corner. Int. J. Precis. Eng. Manuf..

[3] Chua BL, Lee HJ, Ahn DG, Wang Y (2019). A study on activation algorithm of finite elements for three-dimensional transient heat transfer analysis of directed energy deposition process. Int. J. Precis. Eng. Manuf..

[4] Kunimine T, Miyazaki R, Yamashita Y, Funada Y (2020). Effects of laser-beam defocus on microstructural features of compositionally graded WC/Co-alloy composites additively manufactured by multi-beam laser directed energy deposition. Sci. Rep..

[5] Zhang Y, Sahasrabudhe H, Bandyopadhyay A (2015). Additive manufacturing of Ti–Si–N ceramic coatings on titanium. Appl. Surf. Sci..

[6] Lek JY (2018). Understanding the microstructural evolution of cold sprayed Ti–6Al–4V coatings on Ti–6Al–4V substrates. Appl. Surf. Sci..

[7] Guan X, Zhao YF (2020). Modeling of the laser powder-based directed energy deposition process for additive manufacturing: A review. Int. J. Adv. Manuf. Technol..

[8] Izadi M, Farzaneh A, Mohammed M, Gibson I, Rolfe B (2020). A review of laser engineered net shaping (LENS) build and process parameters of metallic parts. Rapid Prototype J..

[9] Kim DH (2018). Smart machining process using machine learning: A review and perspective on machining industry. Int. J. Precis. Eng. Manuf. Green Technol..

[10] Liu S, Shin YC (2019). Additive manufacturing of Ti6Al4V alloy: A review. Mater. Des..

[11] Ahn DG (2021). Directed energy deposition (DED) process: State of the art. Precis. Eng. Manuf. Green Technol..

[12] Qi X, Chen G, Li Y, Cheng X, Li C (2019). Applying neural-network-based machine learning to additive manufacturing: Current applications. Chall. Future Perspect. Eng..

[13] Khanzadeh M, Chowdhury S, Marufuzzaman M, Tschopp MA, Bian L (2018). Porosity prediction: Supervised-learning of thermal history for direct laser deposition. J. Manuf. Syst..

[14] Sreeraj P, Kannan T (2012). Modelling and prediction of stainless steel clad bead geometry deposited by GMAW using regression and artificial neural network models. Adv. Mech. Eng..

[15] Li X, Jia X, Yang Q, Lee J (2020). Quality analysis in metal additive manufacturing with deep learning. J. Intell. Manuf..

[16] Li X, Siahpour S, Lee J, Wang Y, Shi J (2020). Deep learning-based intelligent process monitoring of directed energy deposition in additive manufacturing with thermal images. Proc. Manuf..

[17] Gaikwad A, Giera B, Guss GM, Forien JB, Matthews MJ, Rao P (2020). Heterogeneous sensing and scientific machine learning for quality assurance in laser powder bed fusion—A single-track study. Addit. Manuf..

[18] Feenstra DR, Molotnikov A, Birbilis N (2021). Utilisation of artificial neural networks to rationalise processing windows in directed energy deposition applications. Mater. Des..

[19] Zhan Z, Li H (2021). Machine learning based fatigue life prediction with effects of additive manufacturing process parameters for printed SS 316L. Int. J. Fatigue.

[20] Zhang Y, Hong GS, Ye D, Zhu K, Fuh JYH (2018). Extraction and evaluation of melt pool, plume and spatter information for powder-bed fusion AM process monitoring. Mater. Des..

[21] Gobert C, Reutzel EW, Petrich J, Nassar AR, Phoha S (2018). Application of supervised machine learning for defect detection during metallic powder bed fusion additive manufacturing using high resolution imaging. Addit. Manuf..

[22] Aoyagi K, Wang H, Sudo H, Chiba A (2019). Simple method to construct process maps for additive manufacturing using a support vector machine. Addit. Manuf..

[23] Kobryn PA, Semiatin SL (2003). Microstructure and texture evolution during solidification processing of Ti–6Al–4V. J. Mater. Process. Technol..

[24] Subudhi A, Dash M, Sabut S (2020). Automated segmentation and classification of brain stroke using expectation-maximization and random forest classifier. Biocybern. Biomed. Eng..

[25] Products for Metal Powder. http://www.koswire.com/en/product/kosmetal.asp. Accessed 05 October 2021.

[26] Sampson R, Lancaster R, Sutcliffe M, Carswell D, Hauser C, Barras J (2021). The influence of key process parameters on melt pool geometry in direct energy deposition additive manufacturing systems. Opt. Laser Technol..

[27] Matthew, Jr. & Donachie, J. Heat treating titanium and its alloys. In *Heat Treating Progress*, 47 (2001).

[28] Alcisto J, Enriquez A, Garcia H, Hinkson S, Hahn M, Foyos J, Ogren J, Lee EW, Es-Said OS (2004). The effect of thermal history on the color of oxide layers in titanium 6242 alloy. Eng. Fail. Anal..

[29] Vladimir C, Yunqian M (2004). Practical selection of SVM parameters and noise estimation for SVM regression. Neural Netw..

