# Supplementary material for: Selection of effective manufacturing conditions for directed energy deposition process using machine learning methods
Source: Sci Rep. 2021 Dec 17;11:24169. doi: 10.1038/s41598-021-03622-z (PMC8683500; doi:10.1038/s41598-021-03622-z)
Supplement: Supplementary file 1 — Supplementary Tables. [file 41598_2021_3622_MOESM1_ESM.docx]

**Appendix A**

| Silver | Blue | Deep-Blue |
| --- | --- | --- |
| 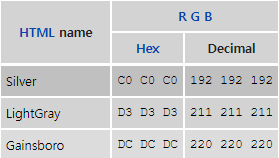 | 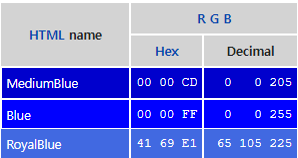 | 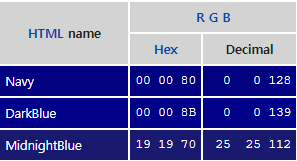 |
| Brown | Gold | Blue-white |
| 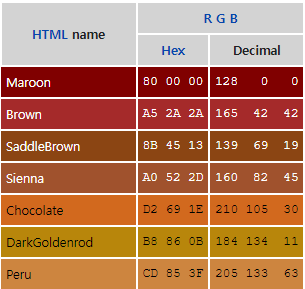 | 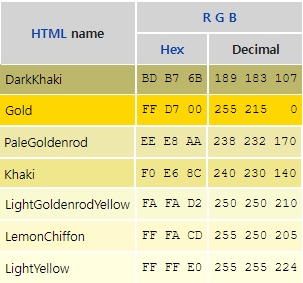 | 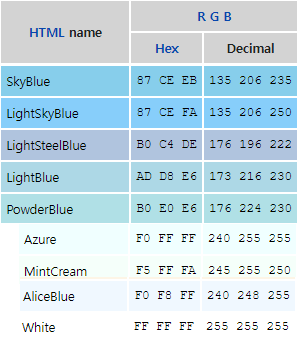 |

**Table A.** Color thresholding (CCS Color Module Level 3, W3c, W3C Recommendation 05 August 2021, https://www.w3.org/TR/css-color-3/ (Accessed 23 November 2021))

**Appendix B**

| LP | SS | Surface color | Vickers hardness | | | | |
| --- | --- | --- | --- | --- | --- | --- | --- |
| 800 | 13 | Gold | 235 | 242 | 302 | 227 | 230 |
| 850 | 13 | Gold | 233 | 248 | 301 | 231 | 233 |
| 900 | 12 | Gold | 235 | 242 | 300 | 230 | 228 |
| 900 | 13 | Gold | 228 | 247 | 307 | 218 | 216 |
| 900 | 13 | Gold | 229 | 246 | 310 | 227 | 210 |
| 850 | 12 | Gold | 230 | 241 | 319 | 234 | 220 |
| 600 | 14 | Gold | 231 | 242 | 299 | 229 | 231 |
| 700 | 14 | Gold | 235 | 243 | 301 | 232 | 229 |
| 800 | 14 | Gold | 233 | 245 | 308 | 222 | 230 |
| 900 | 15 | Gold | 232 | 241 | 306 | 227 | 218 |
| 1000 | 12 | Brown | 280 | 275 | 305 | 228 | 241 |
| 1000 | 13 | Brown | 276 | 221 | 298 | 269 | 239 |
| 1100 | 13 | Brown | 278 | 230 | 301 | 270 | 243 |
| 1000 | 14 | Brown | 283 | 228 | 302 | 233 | 239 |
| 1100 | 14 | Brown | 281 | 271 | 300 | 228 | 248 |
| 1200 | 14 | Brown | 272 | 230 | 296 | 268 | 233 |
| 1200 | 15 | Brown | 266 | 253 | 305 | 239 | 244 |
| 1250 | 16 | Brown | 279 | 242 | 299 | 241 | 241 |
| 1300 | 14 | Brown | 271 | 268 | 298 | 277 | 240 |
| 1300 | 15 | Brown | 269 | 270 | 301 | 242 | 248 |
| 1100 | 12 | Blue | 278 | 308 | 390 | 325 | 275 |
| 1200 | 12 | Blue | 283 | 334 | 350 | 324 | 280 |
| 1300 | 12 | Blue | 280 | 325 | 384 | 312 | 279 |
| 1200 | 13 | Blue | 281 | 311 | 379 | 313 | 280 |
| 1300 | 13 | Blue | 290 | 333 | 365 | 334 | 283 |
| 1400 | 13 | Blue | 287 | 318 | 345 | 326 | 278 |
| 1300 | 14 | Blue | 288 | 328 | 357 | 325 | 275 |
| 1400 | 14 | Blue | 279 | 339 | 351 | 327 | 290 |
| 1500 | 14 | Blue | 291 | 321 | 369 | 312 | 287 |
| 1500 | 15 | Blue | 292 | 330 | 378 | 335 | 291 |
| 1500 | 13 | Blue-White | 341 | 369 | 378 | 370 | 348 |
| 1800 | 14 | Blue-White | 345 | 370 | 388 | 375 | 358 |
| 1900 | 14 | Blue-White | 350 | 375 | 380 | 372 | 359 |
| 2000 | 14 | Blue-White | 347 | 374 | 381 | 371 | 352 |
| 1700 | 15 | Blue-White | 342 | 370 | 379 | 371 | 351 |
| 1800 | 15 | Blue-White | 346 | 368 | 388 | 375 | 350 |
| 1900 | 15 | Blue-White | 345 | 372 | 382 | 380 | 344 |
| 2000 | 15 | Blue-White | 338 | 377 | 380 | 376 | 349 |
| 1500 | 12 | Blue-White | 332 | 372 | 387 | 384 | 350 |
| 1600 | 13 | Blue-White | 342 | 375 | 385 | 370 | 345 |

**Table B.** Hardness measurement results by color of deposition surface.

**Appendix C**

- Gold

| **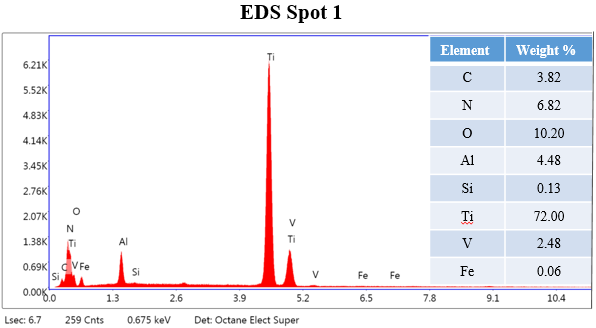** | **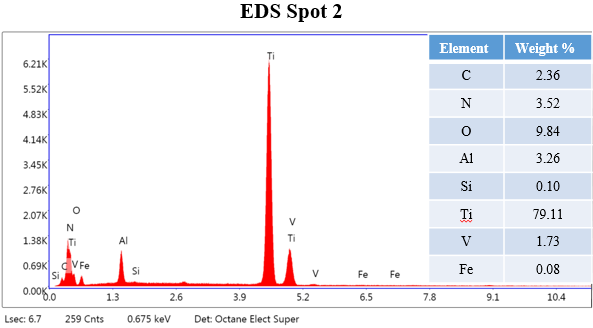** |
| --- | --- |
| **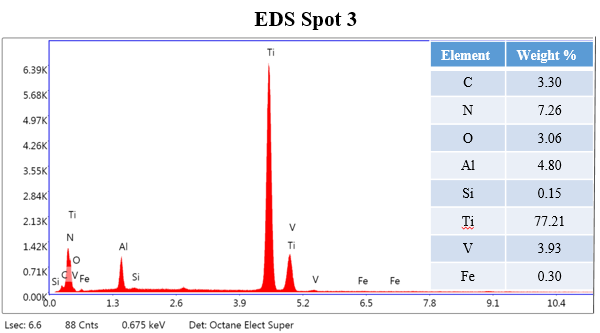** | **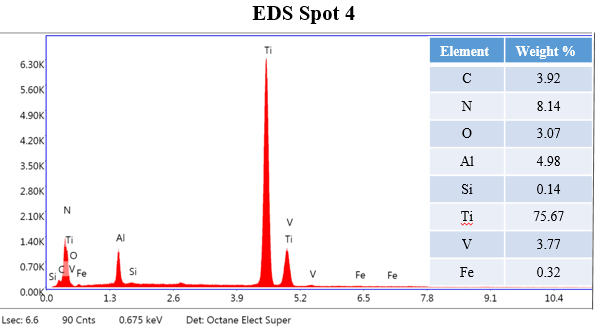** |
| **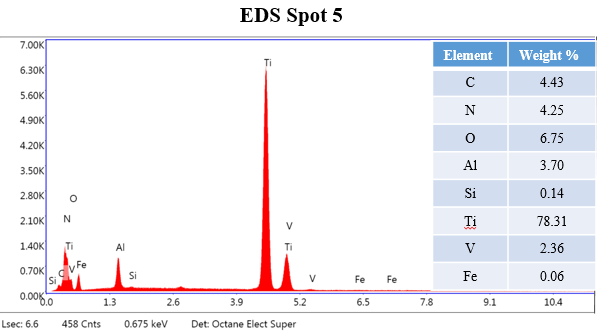** | **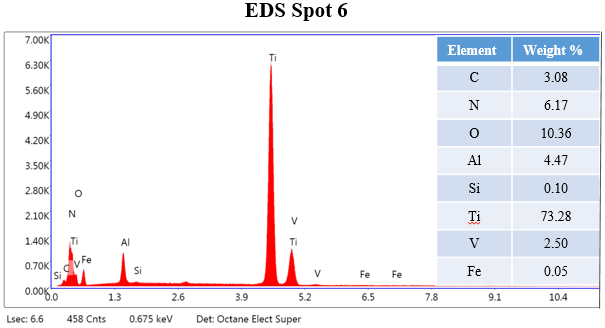** |
| **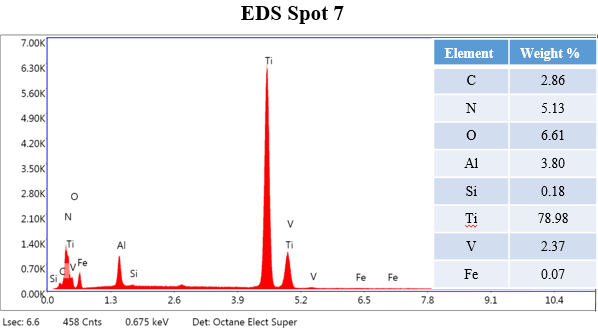** | **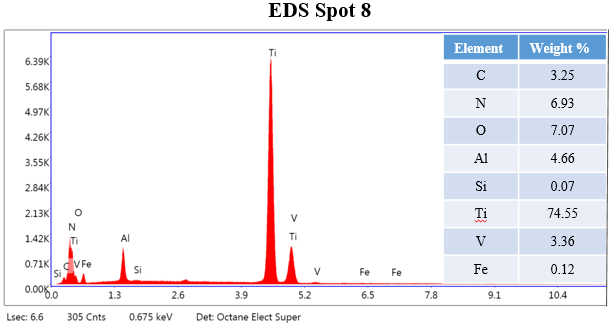** |
| **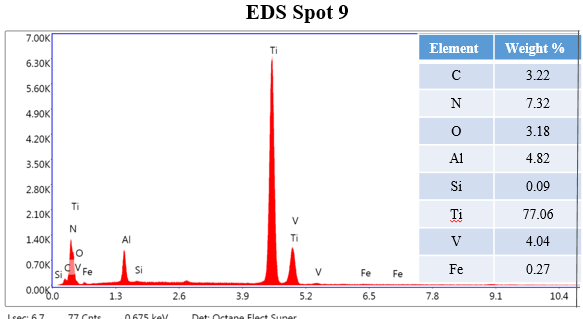** | **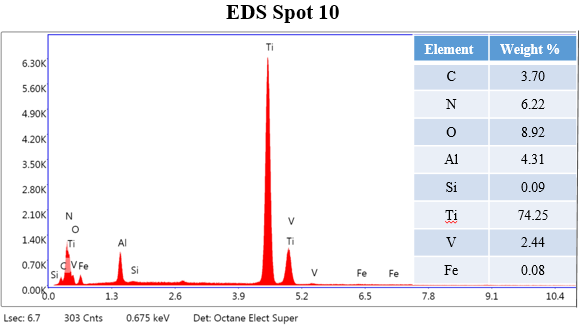** |

- Brown

| **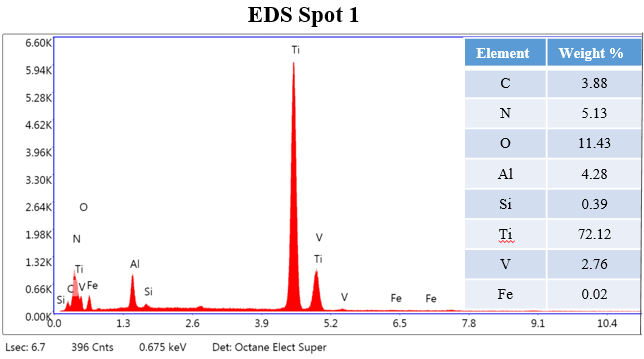** | **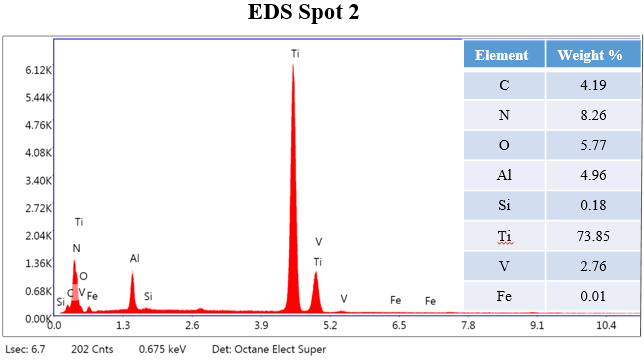** |
| --- | --- |
| **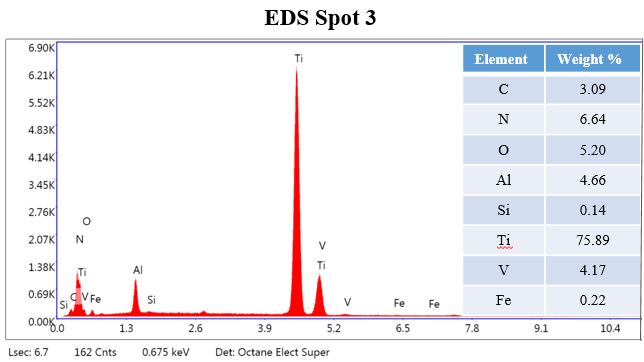** | **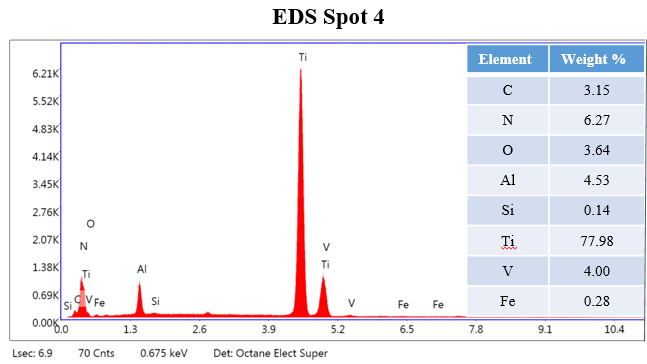** |
| **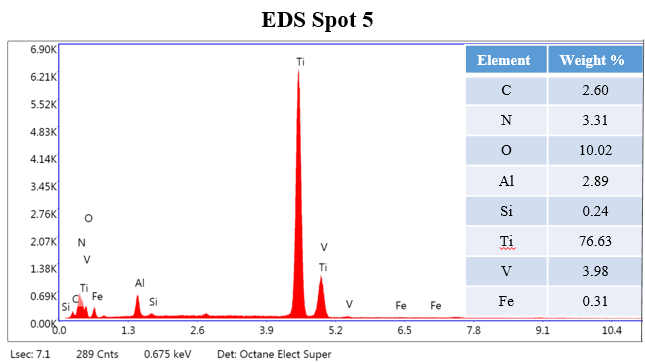** | 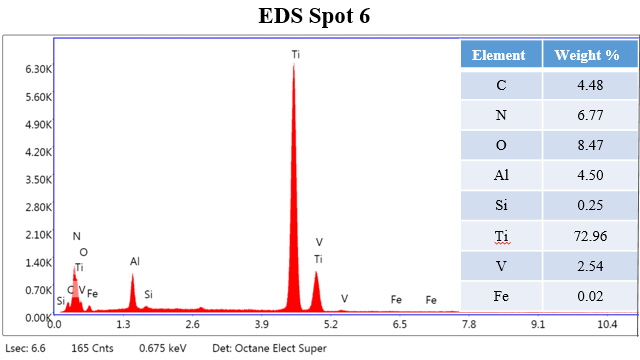 |
| **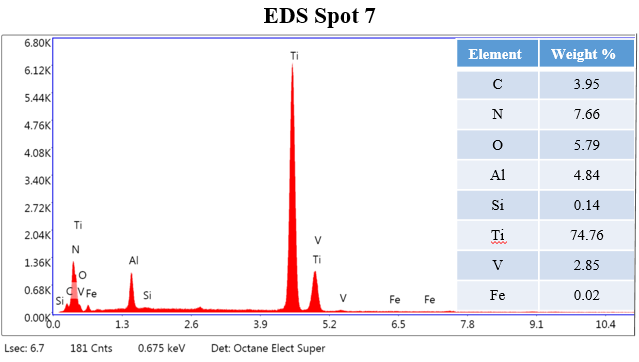** | **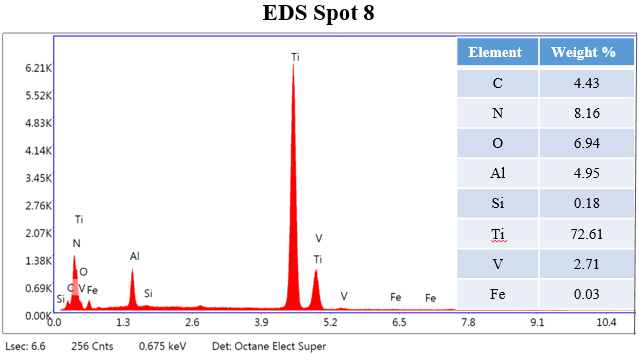** |
| **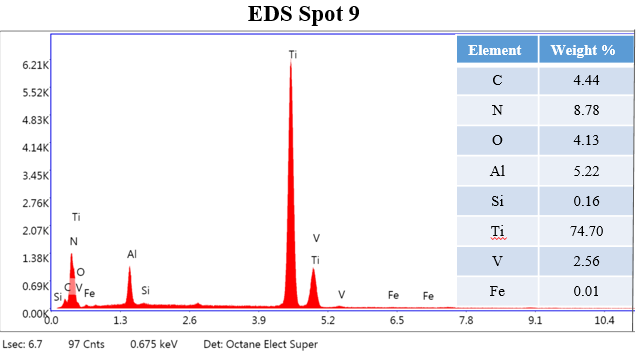** | **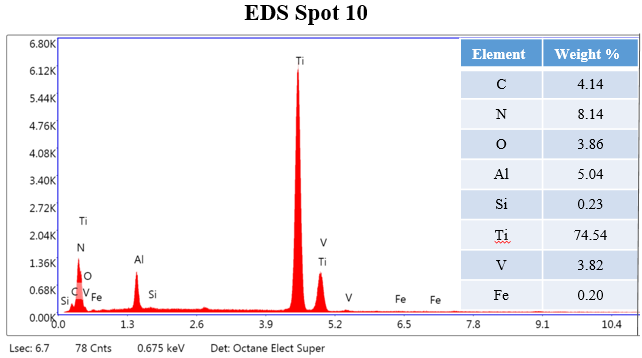** |

- Blue

| **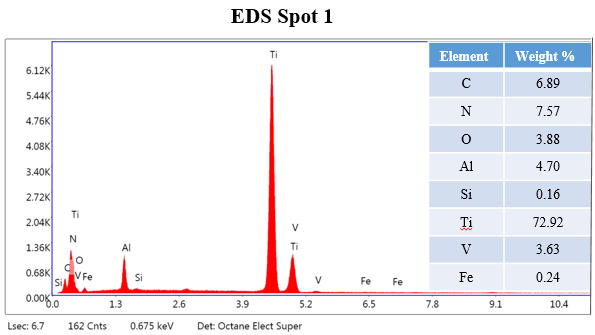** | **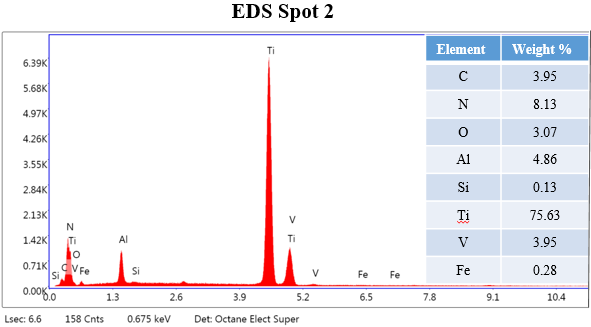** |
| --- | --- |
| **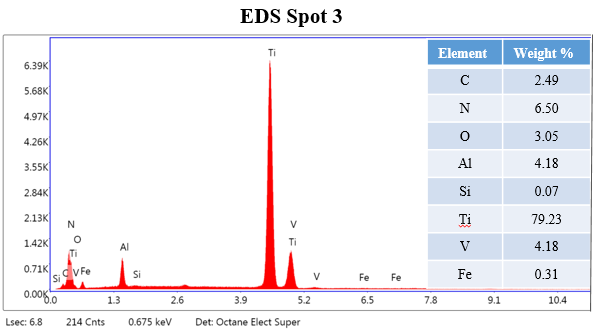** | **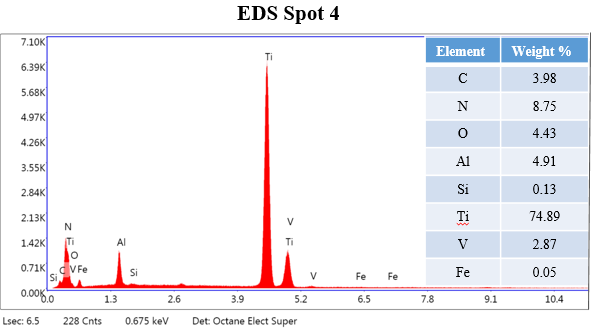** |
| **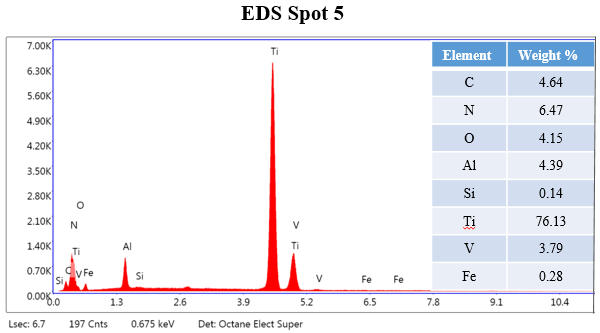** | **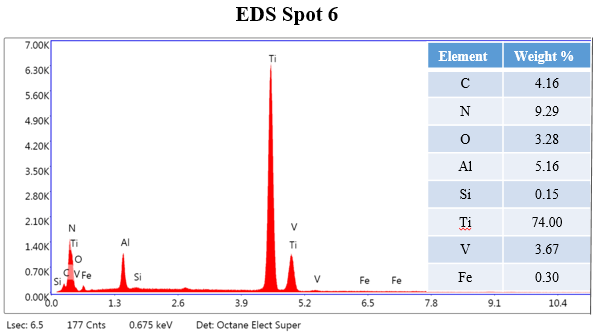** |
| **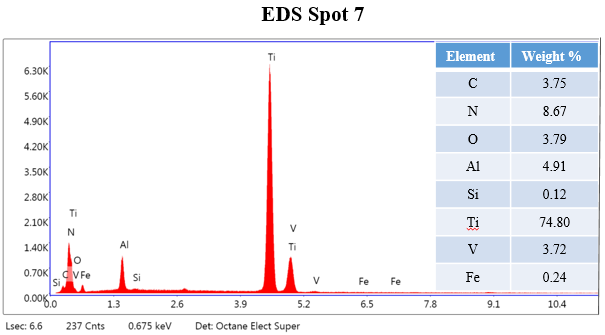** | **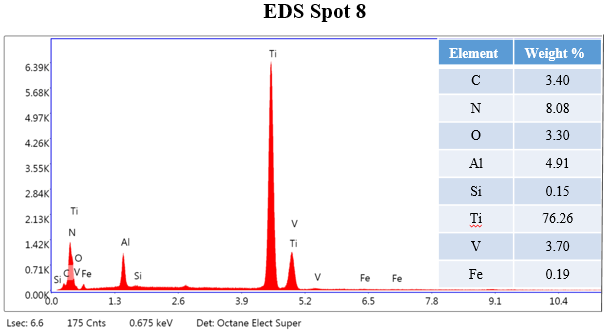** |
| **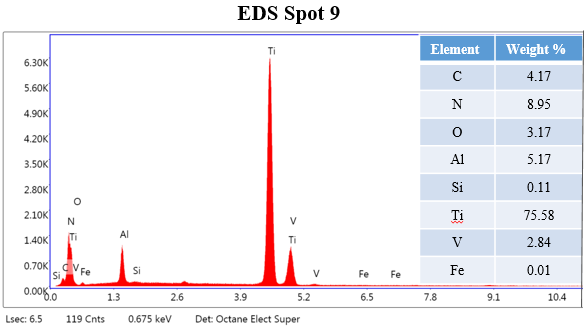** | **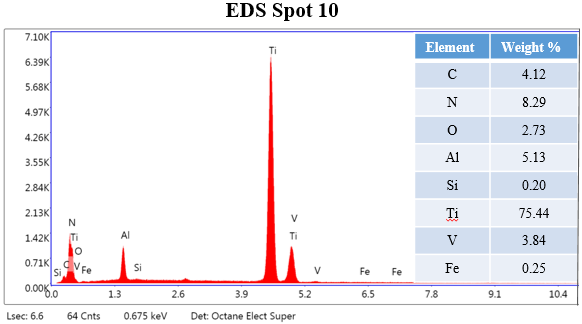** |

- Blue-white

| **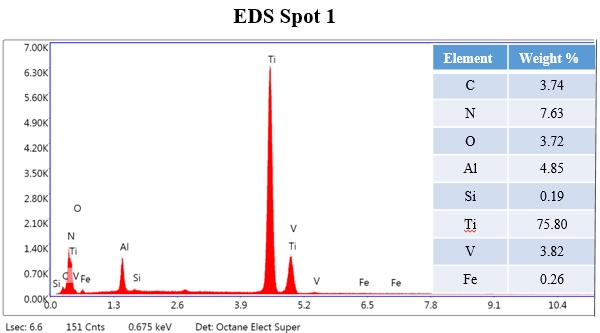** | **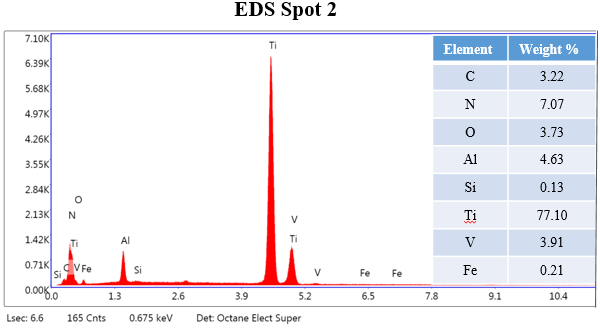** |
| --- | --- |
| **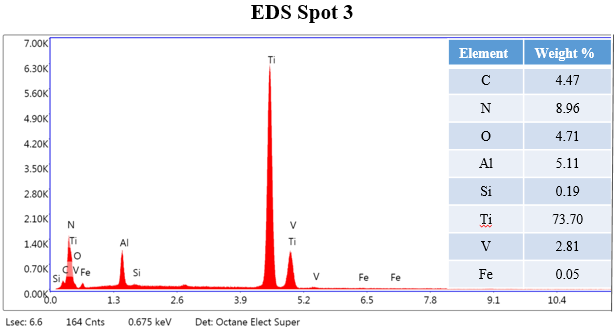** | **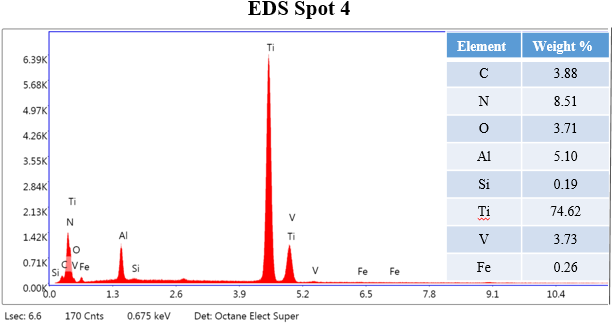** |
| **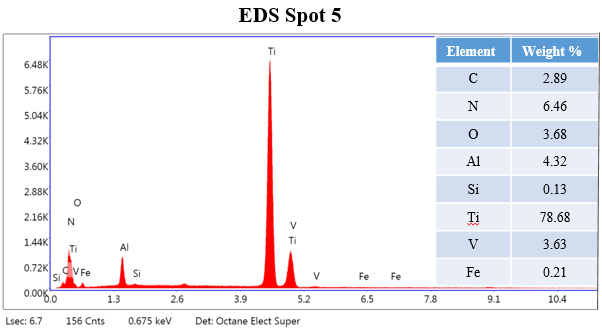** | **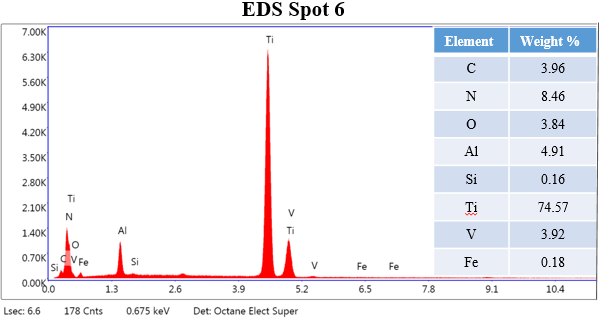** |
| **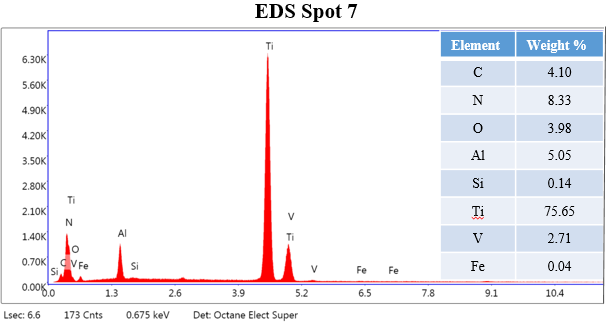** | **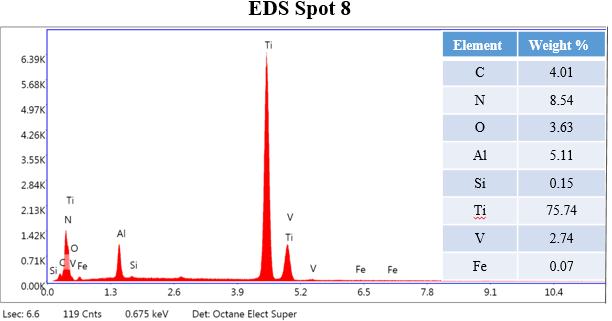** |
| **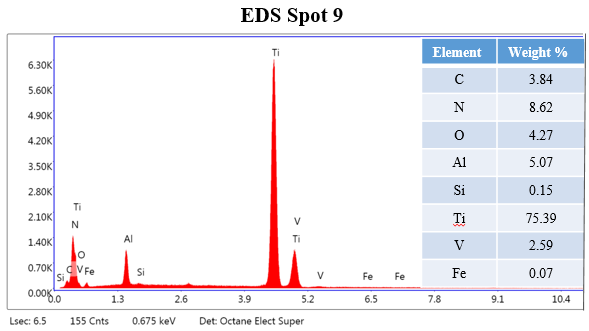** | **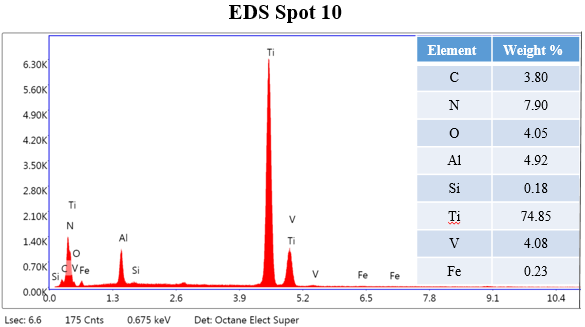** |

**Table C.** EDS component analysis results by color of the deposition surface.
